# Genomic Organisation, Embryonic Expression and Biochemical Interactions of the Zebrafish Junctional Adhesion Molecule Family of Receptors

**DOI:** 10.1371/journal.pone.0040810

**Published:** 2012-07-18

**Authors:** Gareth T. Powell, Gavin J. Wright

**Affiliations:** Wellcome Trust Sanger Institute, Hinxton, Cambridge, United Kingdom; Centro de Investigación en Medicina Aplicada (CIMA), Spain

## Abstract

The mammalian *JAM* family is composed of three cell surface receptors. Interactions between the proteins have well-characterised roles in inflammation and tight junction formation, but little is known about their function in early development. Recently, we identified a role for *jamb* and *jamc* in zebrafish myocyte fusion. Genome duplication in the teleost lineage raised the possibility that additional JAM family paralogues may also function in muscle development. To address this, we searched the zebrafish genome to identify potential paralogues and confirmed their homology, bringing the total number of zebrafish *jam* family members to six. We then compared the physical binding properties of each paralogue by surface plasmon resonance and determined the gene expression patterns of all zebrafish *jam* genes at different stages of development. Our results suggest a significant sub-functionalisation of JAM-B and JAM-C orthologues with respect to binding strength (but not specificity) and gene expression. The paralogous genes, *jamb2* and *jamc2*, were not detected in the somites or myotome of wild-type embryos. We conclude that it is unlikely that the paralogues have a function in primary myogenesis.

## Introduction

The *JAM* (Junctional adhesion molecule) family is a small, deuterostome lineage-restricted subgroup of the immunoglobulin superfamily [1]. There are three *JAM* genes in the human genome, each encoding a type I cell surface receptor with two immunoglobulin domains, a single transmembrane domain and a short cytoplasmic region ending in a C-terminal PDZ domain-binding motif [2]. The members of the family are named *JAM-A*, *JAM-B* and *JAM-C* (official symbols: *F11R*, *JAM2*, *JAM3* respectively; for clarity and consistency with recent literature we use the systematic, unofficial nomenclature, as suggested previously [3]).

To date, the main focus of research into mammalian JAM proteins is their role in inflammation [4]. Heterophilic and homophilic interactions between JAM family proteins are important for their function: JAM-A interacts homophilically [5]–[8] and with JAM-C [9], JAM-B interacts with itself [10] and JAM-C [11]–[14] and JAM-C interacts with itself and JAM-A [9], [12], [13]. To date, no interaction between JAM-B and JAM-A has been demonstrated. Interactions between JAM proteins and integrins, expressed by vascular endothelia and leukocytes, are thought to play a key role in controlling migration of leukocytes into and away from sites of inflammation [5], [9], [11]–[13], [15], [16]. Many other roles have been proposed for each member of the family, including: angiogenesis [17]–[20], cancer [9], [19], [21]–[23], spermatid development and motility [24]–[26], and the maintenance of the myelin sheath formed by Schwann cells [27]. These diverse processes are most likely unified by an important function of the JAM proteins – the formation and maintenance of adhesion and tight junctions between cells [12], [13], [28]–[35].

Although the *JAM*s are known to be expressed during embryogenesis, no developmental function has been described in the various mouse models to date [20], [25], [26], [36]–[40]. Our recent work on cell surface protein interactions identified a function for zebrafish orthologues of JAM-B and JAM-C (named Jamb and Jamc, respectively) in myogenesis [41]. Using cellular transplantation assays, we were able to determine that the heterophilic interaction of Jamb and Jamc between muscle precursor cells (myocytes), *in trans*, was essential for cellular fusion and the formation of normal, syncytial muscle fibres. Loss-of-function of either protein resulted in a potent block in fusion, resulting in an overabundance of mononuclear muscle fibres. The zebrafish orthologue of JAM-A has also been shown to play a role in the development of the lateral line, though the exact function of the protein remains to be elucidated [42].

In *Drosophila*, the cell surface receptors Kirre [43] and Sns [44], and their paralogues Rst [45] and Hbs [46], play a critical role in the development of the larval body wall musculature. The interaction between Kirre and Sns [47] is thought to be the critical step in initiating myocyte fusion between sub-types of precursor cells – founder cells and fusion competent myoblasts (FCMs); the mutually exclusive expression of these proteins in the different sub-types of myocytes is a key regulatory feature [48], [49]. Kirre is expressed by founder cells [43], and Sns is expressed by FCMs [44], so that only founder cell-FCM fusion events can occur. However, loss-of-function of Kirre or Sns alone doesn’t cause a complete loss of fusion. The paralogues of these receptors, Rst and Hbs, play partially redundant functions as they have overlapping expression patterns and are capable of interacting with each other, and Kirre or Sns, to initiate fusion [45]–[47]. The zebrafish genome contains a high number of paralogous genes because of an ancient duplication of the genome in the teleost lineage [50], [51]. The possibility that *jamb* and *jamc* paralogues may also participate in myocyte fusion in zebrafish was of particular interest to us because such a discovery might aid in further elucidating the molecular biology of myogenesis. To address this question, we sought to establish if the *JAM* family had been duplicated in zebrafish and if so, compare the embryonic expression and physical binding properties of the paralogues.

## Results

### Cloning and Homology of Zebrafish Jam Family Members

**Figure 1:**
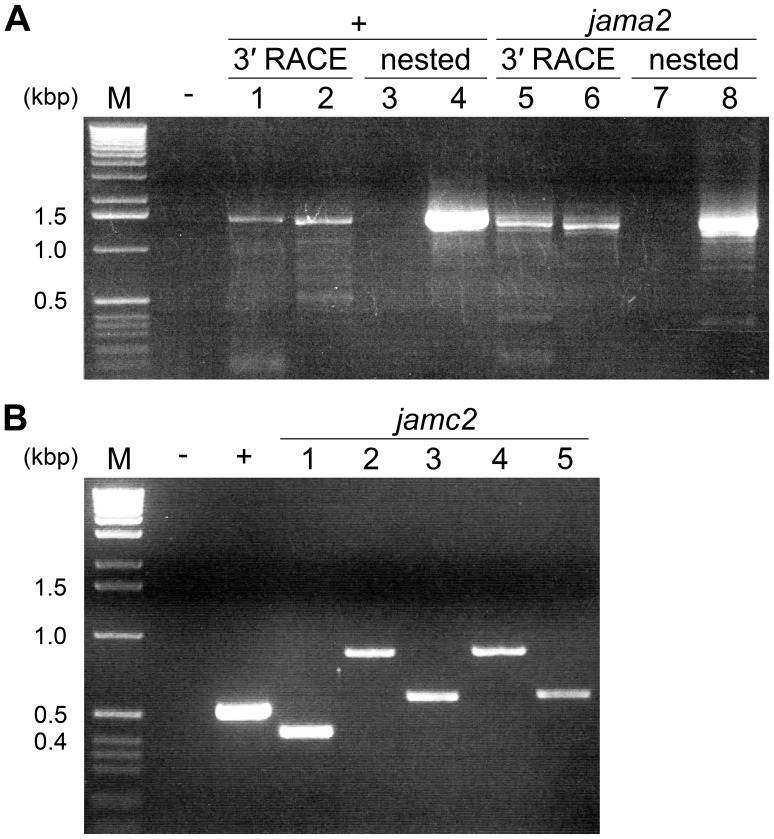
Cloning of putative *jama2 and *jamc2* paralogues by 3′ RACE or RT-PCR.* Agarose gel electrophoresis results of amplification of full-length *jama2* by 3′ RACE (A) or *jamc2* by RT-PCR (B) from cDNA prepared from total RNA extracts of wild-type 24 h. p. f. zebrafish embryos. (A) Lanes: - 3′ RACE negative control; 1–4 *igsf11* positive control: 3′ RACE –1 5′ primer, 2 nested 5′ primer; nested PCR –3 negative control, 4 nested 5′ primer; 5–8 *jama2*∶3′ RACE –5 5′ primer, 6 nested 5′ primer; nested PCR –7 negative control, 8 nested 5′ primer. (B) Lanes: - negative control; + *ef1α* positive control; 1 *jamb* positive control; 2 & 4 *jamc2* primers designed to amplify predicted full-length ORF; 3 & 5 *jamc2* primers designed to amplify IgSF domains. M, DNA size markers with size of selected bands indicated in kbp (left).

Four zebrafish JAM family orthologues, F11r, Jam2a, Jam2b and Jam3b (hereafter referred to as Jama, Jamb, Jamb2 and Jamc respectively; see Materials and Methods for official nomenclature and database entries), were included in recent AVEXIS screens for cell surface protein interactions that are important for zebrafish development [52]–[54]. The interaction between Jamb and Jamc was identified [52] and later found to be essential for myocyte fusion during muscle development [41]. To identify paralogues of *jama* and *jamc* that may have arisen because of genome duplication in the teleost lineage [50], [51], we searched the zebrafish genome using the protein sequence of the extracellular domains of Jama and Jamc, reasoning that the immunoglobulin superfamily (IgSF) domains are likely to be the most conserved amongst JAM family members. We identified and cloned the full length sequences of potential candidates by 3′ RACE or RT-PCR (Figure 1). For clarity, hereafter we refer to these candidates as *jama2* (official symbol *si:ch211-89p1.1*) and *jamc2* (official symbol *jam3a*).

**Figure 2:**
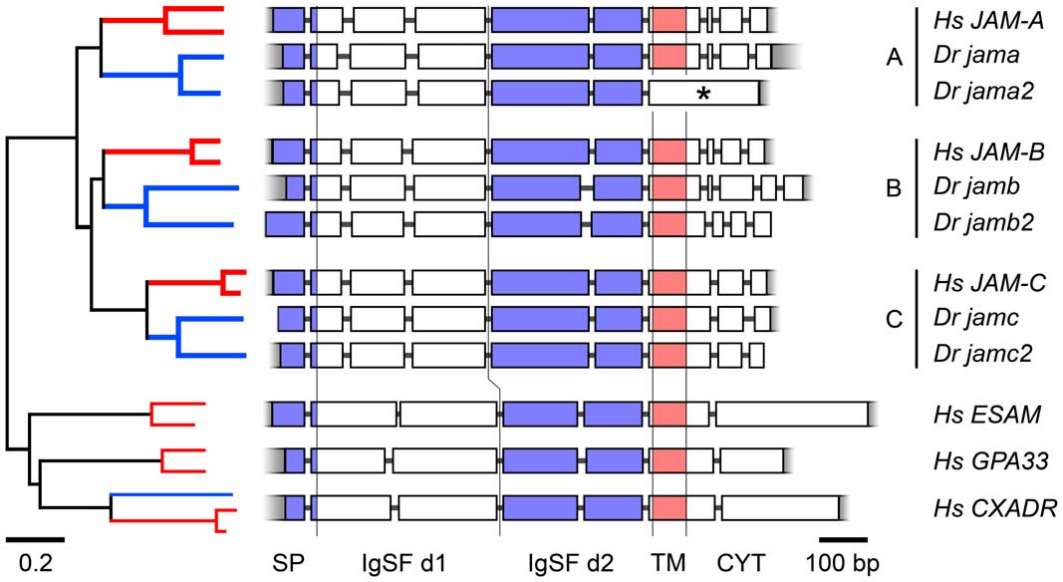
Sequence and gene structure analysis confirms homology to *JAM* family. Phylogenetic tree (left) drawn from protein sequence alignments of the ectodomains of mammalian (human and mouse, red lines) and putative zebrafish (blue lines) *JAM* family orthologues and related cell surface proteins *ESAM*, *GPA33*, and *CXADR*, which serve as an outgroup. Schematic of intron-exon structure of human and zebrafish *JAM* family genes and human *ESAM*, *GPA33*, and *CXADR* (right; drawn to scale). Regions encoding a signal peptide (SP), distal IgSF domain (IgSF d1), proximal IgSF domain (IgSF d2), transmembrane domain (TM) and cytoplasmic domain (CYT) are separated by vertical lines and annotated accordingly. The final coding exon of *jama2* (*) is predicted to encode a coiled-coil motif, but not a transmembrane domain.

To assess the homology of both genes to zebrafish *jama*, *jamc,* and the mammalian orthologues, we took four complementary approaches. Alignments of the ectodomains of zebrafish and mammalian JAM family proteins showed conservation of key features (Figure S1) such as the putative binding motif in the distal IgSF domain (R-V/I/L-E; [7], [8]), or in the case of JAM-B and JAM-C orthologues, the non-canonical disulphide bond between A and G β-strands in the proximal IgSF domain. In addition, five of the zebrafish paralogues are predicted to have a transmembrane domain and a conserved C-terminal type II PDZ-domain binding motif; in Jama2 the cytoplasmic region has been replaced with a single domain predicted to encode a coiled-coil motif (Figure 2).

We constructed a phylogeny from alignments of the protein sequences of the IgSF domains of human, mouse and zebrafish JAM family proteins, using other mammalian two-IgSF-domain-containing cell surface proteins as an outgroup (ESAM, GPA33 and CXADR; Figure 2). Jama2 and Jamc2 were shown to be most closely related to JAM-A and JAM-C orthologues, respectively. We compared the intron-exon structure of human and zebrafish *JAM* family genes and those of the human genes *ESAM*, *GPA33* and *CXADR* (Figure 2 and Figure S1). The zebrafish *JAM* family orthologues were found to have a more similar gene structure to the human *JAM* genes than that of *ESAM*, *GPA33* and *CXADR* – particularly the unusual division of the distal IgSF domain into three exons, but also in the short cytoplasmic domains composed of multiple exons (with the obvious exception of *jama2*).

Finally, we compared the genomic loci of *JAM* family orthologues in human, mouse, chick (where possible; no *JAM-A* orthologue could be identified in the chick genome) and zebrafish (Figure S2). The conservation of presence, orientation and relative position of other genes at these loci in all four genomes demonstrated an evolutionary relationship between the *JAM* family orthologues.

All four analyses supported the conclusion that the putative paralogues, *jama2* and *jamc2* were indeed orthologues of *JAM-A* and *JAM-C*, respectively. Having established the presence of six zebrafish *jam* family genes, we sought to assess if the paralogues shared biochemical properties and gene expression patterns.

### Biochemical Properties of Zebrafish Jam Family Ectodomains

To assess the biochemical properties of each zebrafish JAM family cell surface receptor, we cloned the IgSF-domain-encoding regions of each gene and expressed them as tagged, monomeric proteins using a mammalian expression system [52]. We performed a systematic all-against-all screen using surface plasmon resonance (SPR) to identify and quantify any interactions. Because homophilic interactions within the analyte would lead to an underestimation of analyte activity and consequently an underestimation of the equilibrium binding constant, we chose to measure dissociation rate constants (*k*_d_) as they are independent of analyte activity, assuming first-order kinetics. Previously, we observed a 1∶1 stoichiometry of binding between Jamb and Jamc [41], so we expected that all Jam family interactions we detected would also have first-order dissociation kinetics. These measurements allowed us to compare the relative binding strengths of each interaction between family members. Each analyte was purified and then resolved by gel filtration chromatography immediately before use to ensure the removal of aggregated proteins that can influence the measurement of kinetic parameters. Each ectodomain eluted as a monodisperse peak from the gel filtration column at similar retention volumes.

**Figure 3:**
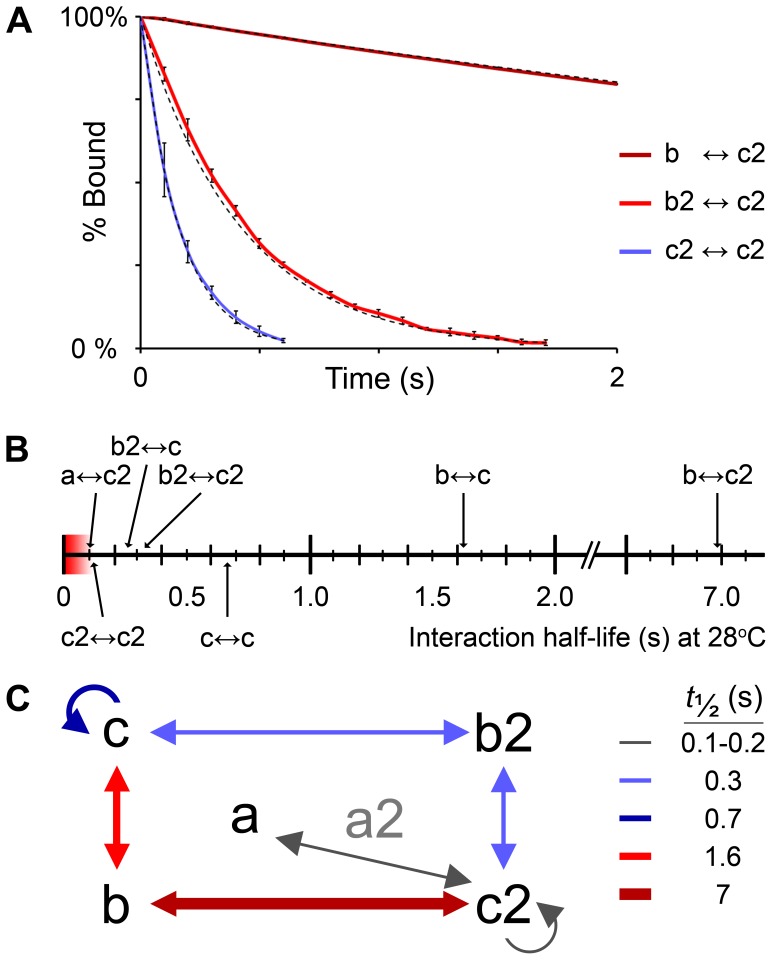
Zebrafish Jam paralogues have similar interaction specificity, but differing binding strengths. (A) Graph showing examples of SPR dissociation phase data from interactions between zebrafish Jam family ectodomains. Coloured lines represent mean of three experiments for a given interaction; error bars represent standard deviation; dashed lines indicate the theoretical first-order decay curve for each interaction shown. (B) Scale bar indicating the average half-life of each heterophilic (above the scale bar) and homophilic (below) interaction that could be quantified (*t*_½_ ≥0.1 s at 28°C). (C) Diagram of interactions (arrows) between zebrafish Jam family ectodomains, coloured according to half-life. The zebrafish JAM-B and JAM-C orthologues show similar specificity of binding, but with differing strengths. Double-headed arrows represent heterophilic interactions tested in both orientations of analyte and ligand; curly arrows represent homophilic interactions. No quantifiable interactions were detected for Jama2 (grey).

We identified 27 interactions amongst family members, within the 36 that we tested (Table S1), with a good fit of the dissociation phase data to a first-order decay curve, again suggesting a 1∶1 binding stoichiometry and validating our assumption of first-order kinetics (Figure 3A). Of these, 16 appeared to have a *t_½_* ≤0.1 s, below the frequency of detection of the instrument used (10 Hz), and could not be reliably quantified. All heterophilic interactions were identified in both orientations of analyte and ligand, with the exception of the weak interaction between Jama and Jama2.

The interactions identified between the zebrafish orthologues closely match the interactions between their mammalian counterparts: JAM-B interacts homophilically [10] and heterophilically with JAM-C [11], [13], [14] and JAM-C also interacts with itself and with JAM-A [9], [13]. We have observed a novel class of interaction: weak heterophilic interactions between the JAM-B orthologues and JAM-A orthologues. Both JAM-A orthologues and both JAM-B orthologues showed the same specificity of binding for other Jam family ectodomains, but only one JAM-C orthologue, Jamc2, was found to interact with both JAM-A orthologues. These results suggest similarity of heterophilic binding specificity between the paralogues. Interestingly, no homophilic interaction was identified for either Jama or Jama2, but they were found to bind each other. In contrast, homophilic interactions of Jamc and Jamc2 were observed, but not a heterophilic interaction between them.

There was a wide range of dissociation constants and half-lives observed amongst the 11 interactions that could be quantified: ∼0.11–7.00 s (average *t_½_* for both orientations of ligand and analyte; Figure 3). The relative strengths of interactions amongst the paralogues were found to be quite different; for example, Jamb bound Jamc with *t_½_* ∼1.63 s, but bound Jamc2 with a much greater *t_½_* ∼7.00 s. Similarly, the homophilic interactions of Jamc and Jamc2 were different: 0.67 s and 0.11 s, respectively. Taken together, these results suggest sub-functionalisation of binding affinity amongst the paralogues, but not binding specificity, as each ectodomain contains the same conserved putative binding motif [7], [8].

### Gene Expression Patterns of Zebrafish Jam Family during Development

**Figure 4:**
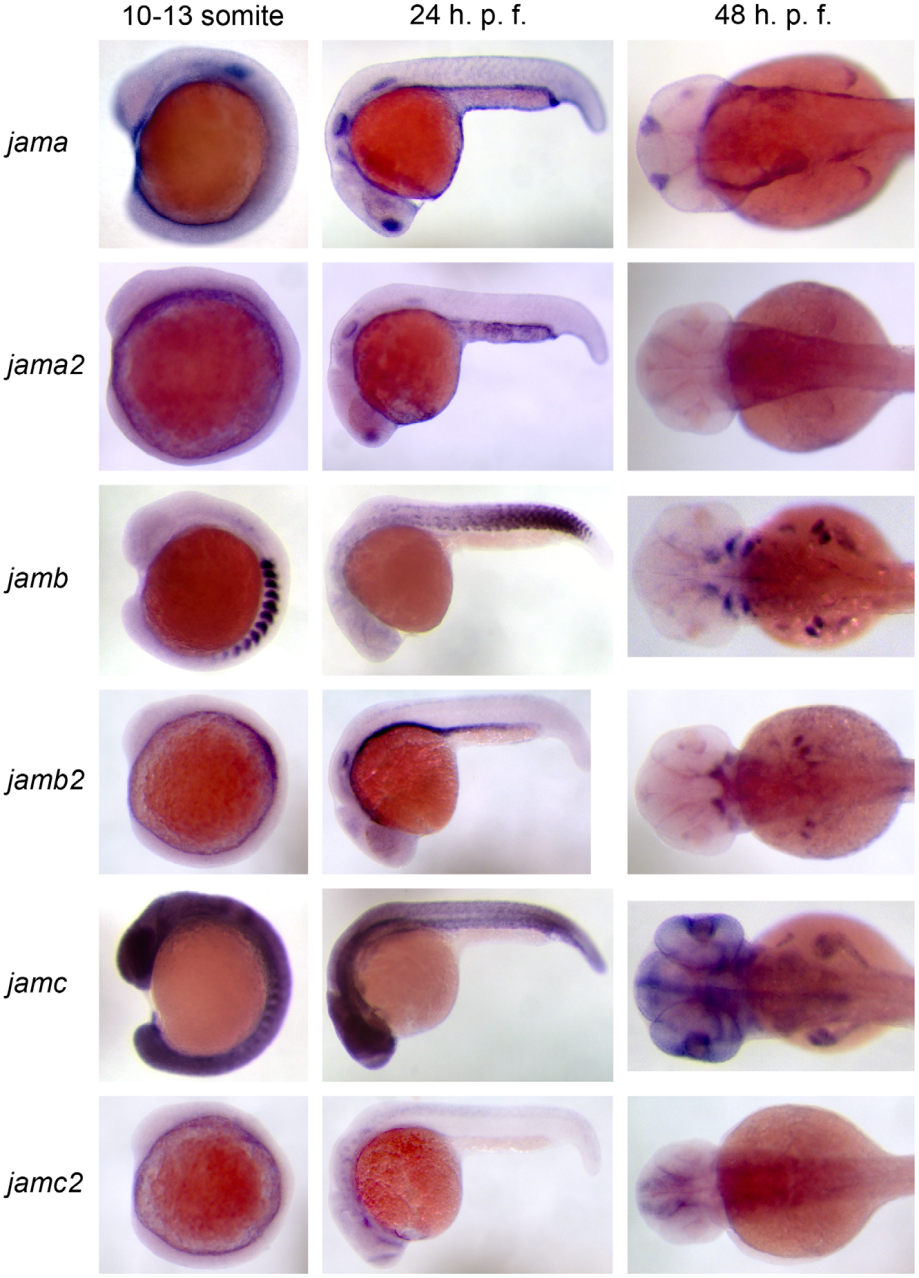
Expression patterns of zebrafish *jam* family genes suggest different transcriptional regulation of paralogues. Wholemount *in situ* hybridisation of *jama* (top row), *jama2* (second row), *jamb* (third row), *jamb2* (fourth row), *jamc* (fifth row) and *jamc2* (bottom row) to wild-type embryos at three different stages of development: approximately 10–13 somites (left column), 24 h. p. f. (middle column) and 48 h. p. f. (right column). *JAM-B* and *JAM-C* orthologues show little overlap in expression during embryogenesis, suggesting different regulation of the paralogues.

To assess any similarity of spatio-temporal gene expression patterns between zebrafish *jam* family paralogues during development, we performed a systematic wholemount in situ hybridisation screen using antisense riboprobes transcribed from the extracellular IgSF-encoding regions of each paralogue. We observed expression in wild-type zebrafish embryos at different stages of development (Figure 4, Table S2): shield (∼6 h. p. f.; data not shown), 10–13 somites (∼14–15½ h. p. f.; [41]), 21 somites (∼19½ h. p. f.; data not shown), 24 h. p. f. [41] and 48 h. p. f. [41].

There was very little similarity between the expression patterns of the *JAM-B* orthologues until 48 h. p. f., where both paralogues were expressed in the pectoral fin; there may also be some overlap in expression in the branchial/mandibular arches and craniofacial mesoderm at this stage. The expression patterns of the *JAM-C* orthologues were also quite different; *jamc2* was predominantly expressed in the neural tube and forebrain, while *jamc* was strongly expressed in the somites and myotomes until 24 h. p. f., and thereafter in the brain. However, *jamc* also appeared to be expressed ubiquitously at low levels, a result that was replicated with a second riboprobe targeted to the 3′ UTR (data not shown). In contrast, both *JAM-A* orthologues are expressed in similar tissues at each stage tested, primarily the otic vesicle, pronephric ducts and lateral line primordium. The similarity in expression may be due to shared regulatory elements in the genome, as both genes are in close proximity on chromosome 5 (Figure S2). However, it may also be due to cross-hybridisation, as the IgSF-domain encoding region of *jama* and *jama2* is highly conserved –82% nucleotide identity by alignment (data not shown). To distinguish between these possibilities, we used antisense riboprobes targeted to the divergent C-terminal domains and 3′ UTRs of either gene. The riboprobe templates were amplified from wild-type cDNA, indicating that both genes are expressed (data not shown). The expression pattern of *jama* was replicated with the C-terminal/3′ UTR probe, but *jama2* expression was not observed (data not shown).

These results show that the transcriptional regulation is different between the paralogues, with the exception of *jama* and *jama2*. We conclude that it is unlikely for *jamb2* and *jamc2* to play a role in muscle development, at least during primary myogenesis.

## Discussion

The duplication of the *JAM* family in the teleost lineage provides a unique opportunity to assess the functions and properties of these genes during vertebrate development. In this study, we identified two additional members of the zebrafish *jam* family, bringing the total number of zebrafish *jam* genes to six. We confirmed their homology – and that of the previously identified genes – to the mammalian orthologues by comparing gene structure, genomic loci and protein features. We then compared the zebrafish paralogues with respect to physical binding properties and gene expression patterns. We observed aspects of sub-functionalisation and innovation within the expanded zebrafish *jam* family.

The *JAM-A* orthologue identified in this study, *jama2*, presents a clear example of innovation. The C-terminal transmembrane domain and cytoplasmic region of *jama* have been replaced with a single exon in *jama2*. This exon is predicted to contain a coiled-coil motif, suggesting that *jama2* is expressed as a secreted multimer. Both paralogues may have similar transcriptional regulation, possibly because of their close proximity in the genome, but this may also be a result of cross-hybridisation – there is a very high degree of identity between the IgSF domains of *jama* and *jama2*, greater than any other pair of zebrafish paralogues and similar to the degree of conservation between human and mouse *JAM*s. We attempted to discriminate between regulation and cross-hybridisation, but were unable to confirm the spatio-temporal expression of *jama2* by in situ hybridisation using a riboprobe targeted to the divergent 3′ end of the transcript. To date, we have been unable to identify a *jama2* homologue in other fish species (stickleback, fugu, medaka and tetraodon) using BLAST searches, suggesting that *jama2* may have arisen from a segmental duplication event unique to zebrafish. In mammals, *JAM*s have been found to play a role in cell polarity [26], [29], [30] and JAM-A has been identified as a component of tight junctions in slit diaphragms – a specialised junction essential for glomerular filtration [55]. The expression of *jama* in the polarised epithelia of the pronephric ducts suggests this may be a function conserved in zebrafish. What function the potentially multimeric, secreted Jama2 protein may have is difficult to evaluate. Soluble forms of JAM-A have been found to increase in inflammation models [56], and soluble forms of JAM-C protein have been linked to angiogenesis [57]. Whether these ectodomains alone have a biological function or are merely a consequence of other processes remains to be ascertained.

Sub-functionalisation within the zebrafish *jam* family is evident when comparing the gene expression patterns and relative binding strengths of the JAM-B and JAM-C orthologues. Protein interaction experiments showed that there is similar specificity of binding amongst the paralogues, but with wide-ranging half-lives: from ∼0.26 s (Jamb2–Jamc) to ∼7 s (Jamb–Jamc2). It is difficult to extrapolate any biological functions for the identified interactions based solely upon relative binding strengths without knowledge of the site of expression of the proteins [58]. Our systematic gene expression analysis suggested that the interaction between JAM-A orthologues and JAM-B orthologues may have a function in the development of the otic vesicle, and interactions between Jamb and either JAM-C orthologue may be relevant in the brain. Of course, homophilic interactions, such as those identified for both JAM-B and JAM-C orthologues, may be biologically relevant in any tissue in which they are expressed. There is little similarity in the timing and distribution of gene expression between the paralogues, suggesting differing transcriptional regulation; this may be because the paralogues are separated onto different chromosomes and are influenced by local transcriptional enhancers.

The results of the protein interaction screen support the conclusion that conservation of the putative binding motif between paralogues retained specificity, but divergence in the binding surface affected relative binding strength. For example, both JAM-C orthologues are capable of binding JAM-B orthologues and interacting homophilically, but are not able to bind each other. In other words, the Jamc paralogues have retained the ability to bind JAM-B orthologues, indicating conservation of the binding site, but lost the ability to bind each other, indicating a divergence in the binding surface. We speculate that heterophilic interactions, *in trans*, might require a different binding surface or binding site to that of homophilic interactions. Our current understanding of the mechanism of interaction between JAM proteins is based upon the structure of homomeric JAM-A dimers, arranged *in cis* in a crystal lattice [7], [8]. Each zebrafish Jam ectodomain contains the conserved putative binding motif but interacts with other family members with different relative strengths. This suggests that changes in the context of the motif between paralogues, the binding surface, are responsible for the heterogeneity of affinity, or indeed, compatibility.

Our main aim was to determine if the zebrafish paralogues of *jamb* and *jamc* might also have a role in early muscle development, as is the case of the *Drosophila* receptor paralogues *kirre*/*rst* [43], [44] and *sns*/*hbs* [45], [46]. We know from our previous study that the interaction between Jamb and Jamc, measured at *t_½_* ∼1.63 s, is physiologically relevant [41]. It remains to be determined if the weaker interactions between the zebrafish paralogues play any role in development. The gene expression patterns of each of the *JAM-B* and *JAM-C* orthologues show that only *jamb* and *jamc* are detectable in the somitic muscle precursors. This makes it unlikely that *jamb2* and *jamc2* have a role in primary myogenesis. However, it remains possible that either gene may be involved in later muscle development, such as that of the craniofacial or pectoral fin musculature.

## Materials and Methods

### Nomenclature and Accession Numbers

We refer to the zebrafish orthologues of *JAM-A*, *JAM-B* and *JAM-C* as *jama*, *jama2*, *jamb*, *jamb2*, *jamc* and *jamc2*, respectively, for the sake of clarity and consistency with other recent literature concerning the *JAM* family [3]. The official symbols and accession/reference numbers are as follows: *jama* (official symbol *f11r*) – Entrez Gene: 323696; *jama2* (official symbol *si:ch211-89p1.1*) – Entrez Gene: 100005566; *jamb* (official symbol *jam2a*) – Entrez Gene: 100005261; *jamb2* (official symbol *jam2b*) – Entrez Gene: 100005301; *jamc* (official symbol *jam3b*) – Entrez Gene: 569217; *jamc2* (official symbol *jam3a*) – Entrez Gene: 797651.

### 3′ RACE and RT-PCR

Zebrafish embryonic RNA was extracted from approximately 30–50 wild-type 24 h. p. f. embryos fixed in methanol, using the Nucleospin RNA II kit (Macherey-Nagal). First strand cDNA was then transcribed from RNA using the SMART RACE kit (Clontech) for *jama2*, or for *jamc2*, using a T_20_VN oligomer and Superscript III reverse transcriptase (Invitrogen). Negative control synthesis reactions, without reverse transcriptase, were prepared in parallel.

Gene-specific nested 5′ primers and SMART RACE universal primers were used to amplify *jama2* by touchdown PCR with Advantage II Polymerase Mix (Clontech). The PCR products were analysed by agarose gel electrophoresis and purified using the QIAquick Gel Extraction kit (QIAGEN). The purified *jama2* 3′ RACE product was cloned into pCR4-TOPO (Invitrogen) and sequenced using the ABI PRISM big dye terminator cycle sequencing ready reaction kit according to the manufacturer’s instructions, in an ABI 3730×1 automatic sequencer.

Different combinations of *jamc2*-specific primers were used in touchdown PCR reactions using 24 h. p. f. wild-type embryo cDNA, and the products were analysed by agarose gel electrophoresis. The full-length product was purified, cloned and sequenced as described above.

### Sequence Alignments and Synteny

Amino acid sequences for mouse and human JAM-A, JAM-B, JAM-C, ESAM, CXADR and GPA33 were retrieved from the NCBI database (www.ncbi.nlm.nih.gov). Alignments of nucleotide and amino acid sequences were performed using ClustalW (www.ebi.ac.uk/ClustalW). A neighbour-joining tree was drawn from this alignment using the Poisson Distribution model in MEGA (v3.1; [59]) using 500 bootstrap replicates. Signal peptide cleavage sites and transmembrane domains were predicted from amino acid sequences using SignalP (v3.0; [60]) and TMHMM (v2.0; [61]).

Ensembl (www.ensembl.org) annotations of all genes within a 500 kbp window centered on a *JAM* family member were manually compared between human, mouse, chick (where possible) and zebrafish genomes for their relative position, strand orientation, exon-intron structure and orthology.

### Protein Production, Purification, and Surface Plasmon Resonance

The extracellular IgSF domains of all zebrafish Jam family members were expressed as a soluble fusion protein with rat Cd4 domains 3 and 4 and either a 6-histidine (Cd4d3+4–6H) or an enzymatically biotinylatable peptide (Cd4d3+4-bio) C-terminal tag. These were purified and used in SPR experiments, essentially as previously described [52]. Dissociation rate constants (*k*_d_) were calculated by averaging the dissociation phase of three different concentrations of purified Jam protein ectodomain and fitting to a simple first-order decay curve. Fits to the data were good, suggesting a 1∶1 stoichiometry of binding. Half lives (*t*_½_) were calculated by *t_½_* = *ln* 2/*k_d_*.

### Zebrafish Husbandry and Embryo Culture

Zebrafish were maintained according to standard fish husbandry conditions and UK Home Office and local ethical regulations and guidelines. Embryos were raised at 28°C in egg water (0.18 g/l sea salt, 2 mg/l methylene blue). Embryos were staged accordingly to morphology, as previously described [62].

### Wholemount in situ Hybridisation

Wholemount in situ hybridisations using digoxygenin-labelled antisense riboprobes were performed using standard protocols [63]. Riboprobe templates were generated from plasmids containing the extracellular IgSF domains of each zebrafish *jam* family member: *jama*, *jama2*, *jamb*, *jamb2*, *jamc* or *jamc2*. Riboprobe templates of the 3′ ends of *jama* and *jama2* were amplified by RT-PCR, using total RNA extracted from wild-type 24 h. p. f. embryos with TRIzol (Invitrogen), as per manufacturer’s instructions.

### Image Acquisition and Processing

Wholemount in situ hybridisation images were obtained using a Zeiss Imager M1 microscope, Zeiss AxioCam Hrc camera, and Zeiss AxioVision software. Entire images were adjusted for contrast, brightness, dynamic range, and resampled to a standardised resolution (300 d. p. i.) using Adobe Photoshop CS2.

## Supporting Information

Figure S1 **Conserved protein features of** ***JAM*** **family ectodomains.** Protein sequence alignments of the extracellular IgSF domains of human, mouse and zebrafish JAM family. Predicted signal peptides are shown in bold text, consensus N-linked glycosylation sites are highlighted in grey, disulphide bridge cysteines in red, alternating black/blue text colour indicates alternate exons and red text indicates codon/exon boundary overlap. Also marked are the putative dimerisation motif highlighted in green and the linker region, VLV residues, underlined and italicised text.
(DOC)

Figure S2 **Conservation of presence, order and orientation of genes between mammalian, avian and zebrafish** ***JAM*** **family loci confirms orthology.** Schematic showing the arrangement and relative position of annotated genes (boxes) within 0.5 Mbp of sequence from human (*Hs*), mouse (*Mm*), chicken (*Gg*) and zebrafish (*Dr*) genomes, centred on *JAM* family orthologues (dark blue boxes): *JAM-A* (A), *JAM-B* (B) and *JAM-C* (C). Genes conserved between mammalian, avian and zebrafish *JAM* family loci are represented in red, except for *JAM-A*, where no chicken homologue could be identified. Genes conserved only between mammalian and avian loci are shown in light blue. Genes indicated in white are unique to each loci. Black boxes (B) indicate *mett121c* paralogues shared between zebrafish JAM-B loci but not present in avian or mammalian loci. Grey box (B) indicates a gene within an intron of the conserved gene *mrpl39* that is not present in avian or mammalian JAM-B loci. Asterisks represent conserved genes present in other loci outside of the 0.5 Mbp window. Drawn to scale.
(TIF)

Table S1 **Dissociation rate constants and half-lives of interactions amongst zebrafish Jam family proteins.** Dissociation rate constant and calculated half-life is presented for each positive interaction observed. Interactions that were too weak to quantify are given the nominal value ≥6.9, equivalent to a half-life of 0.1 seconds. Interactions that could be quantified are presented as a mean ± S. D. (n = 3) and are highlighted in bold; *k*_d_ is presented as a mean ± S. D. (n = 3), *t*_½_ is calculated using the mean *k*_d_ value.
(DOC)

Table S2 **Annotation of zebrafish** ***jam*** **family gene expression patterns during development.** ‘-’ indicates no expression observed; bold indicates major sites of gene expression at each stage.
(DOC)
