# Supplementary material for: Genomic Organisation, Embryonic Expression and Biochemical Interactions of the Zebrafish Junctional Adhesion Molecule Family of Receptors
Source: PLoS One. 2012 Jul 18;7(7):e40810. doi: 10.1371/journal.pone.0040810 (PMC3399880; doi:10.1371/journal.pone.0040810)
Supplement: Figure S1 — Conserved protein features of JAM family ectodomains. Protein sequence alignments of the extracellular IgSF domains of human, mouse and zebrafish JAM family. Predicted signal peptides are shown in bold text, consensus N-linked glycosylation sites are highlighted in grey, disulphide bridge cysteines in red, alternating black/blue text colour indicates alternate exons and red text indicates codon/exon boundary overlap. Also marked are the putative dimerisation motif highlighted in green and the linker region, VLV residues, underlined and italicised text. (DOC) [file pone.0040810.s001.doc]

*Dr* Jama --------------**MVTFAFVCFSLFVTGIHG**-FQVTVTSP-VKVKENEGVDLQCSYTSD 44

*Dr* Jama2 --------------**MVTLVFVCLSFSLTGLHA**SFSVAVNGPVVKVKENEGVDLQCSYTAD 46

*Mm* Jam-A -----**MGTEGKAGRKLLFLFT**-**SMILGSLVQG**KGSVYTAQSDVQVPENESIKLTCTYSG- 53

*Hs* JAM-A -----**MGTKAQVERKLLCLFILAILLCSLALG**SVTVHSSEPEVRIPENNPVKLSCAYSG- 54

*Dr* Jamb --------------**MVVCVSLLILIHSVPVSP**VTVSSRN-PKVEVHEFSDAELSCEFKTE 45

*Dr* Jamb2 **MVLQQPYITKMKTKQLLTSALLLLIYIPSSDP**VTVTTSK-AKVDVHENTNAVLSCEFRTE 59

*Mm* Jam-B ----**MARSPQGLLMLLLLHYLIVALDYHKANG**FSASKDHRQEVTVIEFQEAILACKT-PK 55

*Hs* JAM-B ----**MARRSRHRLLLLLLRYLVVALGYHKAYG**FSAPKDQ-QVVTAVEYQEAILACKT-PK 54

*Dr* Jamc **MALTP**----------**LACVLLLLSMQCYISTLA**VLLKSTNSKPWVNEFESIELSCMIESI 50

*Dr* Jamc2 **MAFGR**----------**QTLSLVLFCWLCNSAAFA**VILRTTEKSVWANEFESIELTCLIESI 50

*Mm* Jam-C **MALSRRLRLRLYARLPDFFLLLLFRGCMIE**--**A**VNLKSSNRNPVVHEFESVELSCIITDS 58

*Hs* JAM-C **MALRRPPRLRLCARLPDFFLLLLFRGCLIG**--**A**VNLKSSNRTPVVQEFESVELSCIITDS 58

* * *

*Dr* Jama FGATPRVEWKFKDLKGSQTLVYFDGKPTGQYTGRVTMYD-KGLRFNKVTRADTGDYDCEV 103

*Dr* Jama2 FGATPRVEWKFRNLKGFQYFIYFNNKPTVEYEQRITVYA-GGLRFQKVTRADAGDYNCEV 105

*Mm* Jam-A F-SSPRVEWKFV-QGSTTALVCYNSQITAPYADRVTFSS-SGITFSSVTRKDNGEYTCMV 110

*Hs* JAM-A F-SSPRVEWKFD-QGDTTRLVCYNNKITASYEDRVTFLP-TGITFKSVTREDTGTYTCMV 111

*Dr* Jamb KDTNPRIEWKRKDKEKDVSFVYYGERFVGPFQDRADIEG-ATVRLRRVTQADAGEYRCEV 104

*Dr* Jamb2 KETNPRVEWKKRGK--DVSYVYFEGDFTGSYKGRASIDG-ATLTLRGVTQKDSGVYHCEV 116

*Mm* Jam-B KTTSSRLEWKKVGQ--GVSLVYYQQALQGDFKDRAEMID-FNIRIKNVTRSDAGEYRCEV 112

*Hs* JAM-B KTVSSRLEWKKLGR--SVSFVYYQQTLQGDFKNRAEMID-FNIRIKNVTRSDAGKYRCEV 111

*Dr* Jamc TTTKPRIEWKKIKNG-DPSYVYFDNQISGDLERRAKIREPATLVILNATRSDSADYRCEV 109

*Dr* Jamc2 STNNPRIEWKKIKNG-VPSYVYFQNKISGDLEHRALLREPANLLILNASRSDTAQYRCEV 109

*Mm* Jam-C QTSDPRIEWKKIQDG-QTTYVYFDNKIQGDLAGRTDVFGKTSLRIWNVTRSDSAIYRCEV 117

*Hs* JAM-C QTSDPRIEWKKIQDE-QTTYVFFDNKIQGDLAGRAEILGKTSLKIWNVTRRDSALYRCEV 117

.*:*** : : * . : : .:: * . * * *

*Dr* Jama SGSGG----YGENTIKLT*VLV*PPAKPVSRIPSSVTTSSNVRLTCFDPVGSPPSTYKWYKD 159

*Dr* Jama2 SGNGG----YGENTIKLV*VSV*PPSKPVSSIPSSVTTGSNVRLTCFDPVGSPPSTYEWYKD 161

*Mm* Jam-A SEEGGQ--NYGEVSIHLT*VLV*PPSKPTISVPSSVTIGNRAVLTCSEHDGSPPSEYSWFKD 168

*Hs* JAM-A SEEGGN--SYGEVKVKLI*VLV*PPSKPTVNIPSSATIGNRAVLTCSEQDGSPPSEYTWFKD 169

*Dr* Jamb SAPSDS-ISLGETNVTLR*VLV*PPQTPSCDVPSSALTGSQVELRCRDRHSIPPAVYTWYKD 163

*Dr* Jamb2 TARQDK-IKLGEVSVTLS*VLV*PPHAPTCEVPEAVMRGFSAELHCKDKLSVPAATYSWYKD 175

*Mm* Jam-B SAPTEQGQNLQEDKVMLE*VLV*APAVPACEVPTSVMTGSVVELRCQDKEGNPAPEYIWFKD 172

*Hs* JAM-B SAPSEQGQNLEEDTVTLE*VLV*APAVPSCEVPSSALSGTVVELRCQDKEGNPAPEYTWFKD 171

*Dr* Jamc TAPNDQ-KSFDEILISLT*VRV*KPVVPRCSVPKSIPVGKPAELHCLEDEGYPKSQYQWFRN 168

*Dr* Jamc2 AAIDDQ-KPFDEILISLA*VRV*KPVIPRCSVPDAVNVGSSTELRCIENEGFPQSQYQWFKN 168

*Mm* Jam-C VALNDR-KEVDEITIELI*VQV*KPVTPVCRIPAAVPVGKTATLQCQESEGYPRPHYSWYRN 176

*Hs* JAM-C VARNDR-KEIDEIVIELT*VQV*KPVTPVCRVPKAVPVGKMATLHCQESEGHPRPHYSWYRN 176

* : * * * * * :* : . . * * : . * . * *:::

*Dr* Jama NTPLPE-DPTKFPAFKNLTYKMNVFNGNLEFPSVSKMDTGSYFCEASNGEGVPQRGDEVK 218

*Dr* Jama2 NNLLPE-DPTKFPIFKNLTYKMNAFNGNLEFLSVSKWDAGSYFCVASNENGVSQHGDAVK 220

*Mm* Jam-A GISMLTADAKKTRAFMNSSFTIDPKSGDLIFDPVTAFDSGEYYCQAQNGYGTAMRSEAAH 228

*Hs* JAM-A GIVMPT-NPKSTRAFSNSSYVLNPTTGELVFDPLSASDTGEYSCEARNGYGTPMTSNAVR 228

*Dr* Jamb NRALP------IRHPN-ATYTVNEFTGVLMFQTVSRSDAGQYHCEAKNGVGPPKSCQHTH 216

*Dr* Jamb2 NKPLN------TANPHDVHYTLDTKTGSLKFKSVSKSDEGQYRCEASNGVGAPKSCAGHH 229

*Mm* Jam-B GTSLLG-NPKGGTHN-NSSYTMNTKSGILQFNMISKMDSGEYYCEARNSVG-HRRCPGKR 229

*Hs* JAM-B GIRLLE-NPRLGSQSTNSSYTMNTKTGTLQFNTVSKLDTGEYSCEARNSVG-YRRCPGKR 229

*Dr* Jamc KEEIPL-DPKSSPKFFNSTYTLDGEMGTLKFSAVRKEDAGEYYCRAKNEAG-ISECGPQM 226

*Dr* Jamc2 SEELPE-DPKTSSKFYNSSYIMNIETGSLKFRSVKKEDAGEYYCQARNEAG-WSKCIRQS 226

*Mm* Jam-C DVPLPT-DSRANPRFQNSSFHVNSETGTLVFNAVHKDDSGQYYCIASNDAG-AARCEGQD 234

*Hs* JAM-C DVPLPT-DSRANPRFRNSSFHLNSETGTLVFTAVHKDDSGQYYCIASNDAG-SARCEEQE 234

: : :: * * * : * *.* * * * *

*Dr* Jama MEVRDLN---- 225

*Dr* Jama2 MEVYDVDSS-- 229

*Mm* Jam-A MDAVELNVG-- 237

*Hs* JAM-A MEAVERNVGV- 238

*Dr* Jamb MQIDDLNVAA- 226

*Dr* Jamb2 MKITEFELNMT 240

*Mm* Jam-B MQVD------- 233

*Hs* JAM-B MQVDDLNIS-- 238

*Dr* Jamc MEVYDINIAG- 236

*Dr* Jamc2 MEVYDLDIV-- 235

*Mm* Jam-C MEVYDLNIAG- 244

*Hs* JAM-C MEVYDLNIGG- 244

*.
