# Supplementary figures and images for: Genomic Organisation, Embryonic Expression and Biochemical Interactions of the Zebrafish Junctional Adhesion Molecule Family of Receptors
Source: PLoS One. 2012 Jul 18;7(7):e40810. doi: 10.1371/journal.pone.0040810 (PMC3399880; doi:10.1371/journal.pone.0040810)

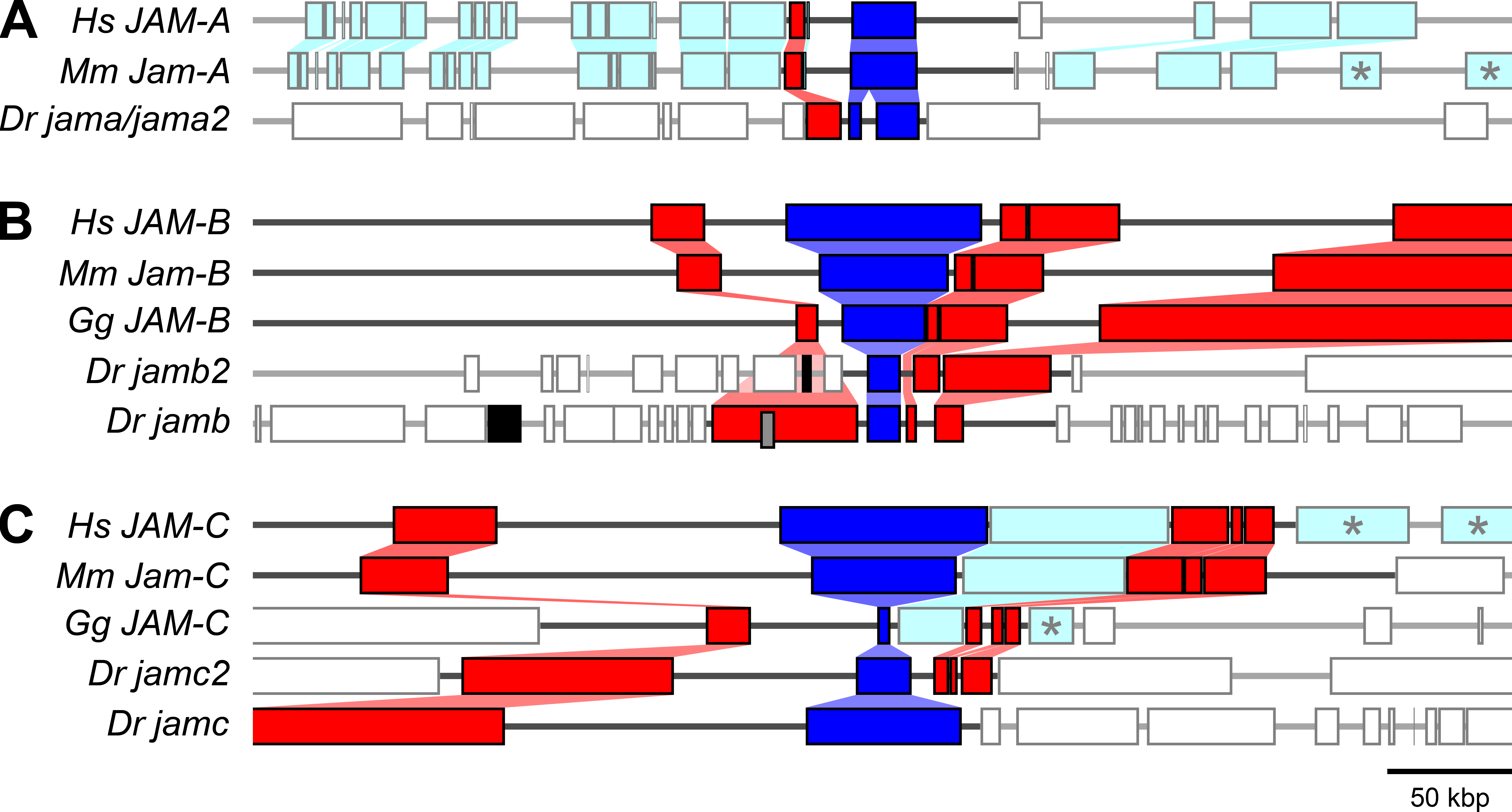

Supplement: Figure S2 — Conservation of presence, order and orientation of genes between mammalian, avian and zebrafish JAM family loci confirms orthology. Schematic showing the arrangement and relative position of annotated genes (boxes) within 0.5 Mbp of sequence from human (Hs), mouse (Mm), chicken (Gg) and zebrafish (Dr) genomes, centred on JAM family orthologues (dark blue boxes): JAM-A (A), JAM-B (B) and JAM-C (C). Genes conserved between mammalian, avian and zebrafish JAM family loci are represented in red, except for JAM-A, where no chicken homologue could be identified. Genes conserved only between mammalian and avian loci are shown in light blue. Genes indicated in white are unique to each loci. Black boxes (B) indicate mett121c paralogues shared between zebrafish JAM-B loci but not present in avian or mammalian loci. Grey box (B) indicates a gene within an intron of the conserved gene mrpl39 that is not present in avian or mammalian JAM-B loci. Asterisks represent conserved genes present in other loci outside of the 0.5 Mbp window. Drawn to scale. (TIF) [file pone.0040810.s002.tif]
