# Supplementary material for: Genomic Organisation, Embryonic Expression and Biochemical Interactions of the Zebrafish Junctional Adhesion Molecule Family of Receptors
Source: PLoS One. 2012 Jul 18;7(7):e40810. doi: 10.1371/journal.pone.0040810 (PMC3399880; doi:10.1371/journal.pone.0040810)
Supplement: Table S1 — Dissociation rate constants and half-lives of interactions amongst zebrafish Jam family proteins. Dissociation rate constant and calculated half-life is presented for each positive interaction observed. Interactions that were too weak to quantify are given the nominal value ≥6.9, equivalent to a half-life of 0.1 seconds. Interactions that could be quantified are presented as a mean ± S. D. (n = 3) and are highlighted in bold; k d is presented as a mean ± S. D. (n = 3), t ½ is calculated using the mean k d value. (DOC) [file pone.0040810.s003.doc]

| **Table S1.** Dissociation rate constants and half-lives of interactions amongst zebrafish Jam family proteins. Dissociation rate constant and calculated half-life of each positive interaction observed. Interactions that were too weak to quantify are given the nominal value ≥ 6.9 s^-1^, equivalent to a half-life of ≥ 0.1 s. Interactions that could be quantified are highlighted in bold; *k*_d_ is presented as a mean ± S. D. (n = 3), *t*_½_ is calculated using the mean *k*_d_ value. | | | | | | | | | | | | |
| --- | --- | --- | --- | --- | --- | --- | --- | --- | --- | --- | --- | --- |
| Ligand | Analyte | | | | | | | | | | | |
|  | Jama | | Jama2 | | Jamb | | Jamb2 | | Jamc | | Jamc2 | |
|  | *k*_d_ (s^-1^) | *t*_½_ (s) | *k*_d_ (s^-1^) | *t*_½_ (s) | *k*_d_ (s^-1^) | *t*_½_ (s) | *k*_d_ (s^-1^) | *t*_½_ (s) | *k*_d_ (s^-1^) | *t*_½_ (s) | *k*_d_ (s^-1^) | *t*_½_ (s) |
| Jama | - | - | - | - | ≥ 6.9 | ≤ 0.1 | ≥ 6.9 | ≤ 0.1 | - | - | **4.43 ± 0.95** | **0.16** |
| Jama2 | ≥ 6.9 | - | - | - | ≥ 6.9 | ≤ 0.1 | ≥ 6.9 | ≤ 0.1 | - | - | ≥ 6.9 | ≤ 0.1 |
| Jamb | ≥ 6.9 | ≤ 0.1 | ≥ 6.9 | ≤ 0.1 | ≥ 6.9 | ≤ 0.1 | ≥ 6.9 | ≤ 0.1 | **0.37 ± <0.01** | **1.87** | **0.09 ± <0.01** | **7.70** |
| Jamb2 | ≥ 6.9 | ≤ 0.1 | ≥ 6.9 | ≤ 0.1 | ≥ 6.9 | ≤ 0.1 | ≥ 6.9 | ≤ 0.1 | **2.44 ± 0.28** | **0.28** | **1.88^†^ ± 0.05** | **0.37** |
| Jamc | - | - | - | - | **0.50 ± 0.04** | **1.39** | **2.86 ± 0.22** | **0.24** | **1.03 ± 0.09** | **0.67** | - | - |
| Jamc2 | ≥ 6.9 | ≤ 0.1 | ≥ 6.9 | ≤ 0.1 | **0.11 ± <0.01** | **6.30** | **2.39^†^ ± 0.30** | **0.29** | - | - | **6.22 ± 0.60** | **0.11** |
| † denotes an interaction that appears to display a two-phase dissociation – the dissociation rate constant is estimated from the first 0.9–1 seconds of dissociation (accounting for approximately 85–90% of specific binding) that fits a first order exponential decay model with an R² ≥ 0.97. | | | | | | | | | | | | |
