# Supplementary material for: Genomic Organisation, Embryonic Expression and Biochemical Interactions of the Zebrafish Junctional Adhesion Molecule Family of Receptors
Source: PLoS One. 2012 Jul 18;7(7):e40810. doi: 10.1371/journal.pone.0040810 (PMC3399880; doi:10.1371/journal.pone.0040810)
Supplement: Table S2 — Annotation of zebrafish jam family gene expression patterns during development. ‘-’ indicates no expression observed; bold indicates major sites of gene expression at each stage. (DOC) [file pone.0040810.s004.doc]

| **Table S2.** Annotation of zebrafish *jam* family gene expression patterns during development. ‘-’ indicates no expression observed, bold indicates major sites of gene expression at each stage. | | | | | |
| --- | --- | --- | --- | --- | --- |
| Gene | Developmental stage | | | | |
|  | Shield | 10-13 somites | 21 somites | 24 h. p. f. | 48 h. p. f. |
| *jama* | **uniform** | **otic placode,**  nasal epithelium | **otic vesicle,**  **nasal epithelium,**  **lateral line primordium,**  **pronephric ducts,**  epidermis,  eye | **otic vesicle,**  **nasal epithelium,**  **lateral line primordium,**  **pronephric ducts,**  epidermis | **otic vesicle,**  **nasal epithelium,**  **lateral line primordium,**  **pronephric ducts,**  epidermis,  pectoral fin |
| *jama2* | **uniform** | - | otic vesicle,  nasal epithelium,  epidermis,  pronephric ducts,  eye | **otic vesicle,**  **nasal epithelium,**  epidermis,  pronephric ducts,  lateral line primordium | otic vesicle,  nasal epithelium,  pectoral fin |
| *jamb* | - | **posterior epithelium and mesenchyme of mature somites,**  anterior and posterior poles of otic placode | **myotome/mature somites, isfidfififififbxcbcvbcvifif**  anterior and posterior poles of otic vesicle,  forebrain | **caudal somites,** nsdfhskfhkhskdfhscncncncshsthththrhrthnc  otic vesicle, ekfhasdkfhkfhsdkfjhsdakfjhasdkfjh  brain | **pectoral fin muscles,**  **hypaxial/epaxial muscles,**  **craniofacial mesoderm** |
| *jamb2* | - | **epithelium over yolk ball** | **epithelium over yolk ball,**  otic vesicle | **epithelium over yolk ball,**  otic vesicle | **pectoral fin muscles,**  **branchial / mandibular arches** |
| *jamc* | - | **posterior-medial foci in rostral somites,**  fawuihfauifhsfuhsufhafhas  hindbrain, sdflndfklnsdlkfnsd  ubiquitous expression | **dorsal/ventral myotome, dfzdfhdfhfh**  **posterior-medial foci caudal somites,**  sdofizxfhsdfhsdifhsdifhsdifh  ubiquitous expression | **caudal somites,** dslkncsncrthsrhrhtrhthsrhthtrhthtdsl  **eye,** et4asergerrgergryse  **hindbrain /forebrain,**  ubiquitous expression | **hypaxial/epaxial muscles, fksdfkjfhsdfjshf**  **pectoral fin muscles,**  **craniofacial mesoderm,**  ubiquitous expression,  eye |
| *jamc2* | - | - | **neural tube,**  anterior lateral line primordium | **neural tube,**  **brain** | **brain** |
